# In Vivo Validation of a Nanostructured Electrospun Polycaprolactone Membrane Loaded with Gentamicin and Nano-Hydroxyapatite for the Treatment of Periodontitis

**DOI:** 10.3390/membranes14030060

**Published:** 2024-02-26

**Authors:** Patricia Ondine Lucaciu, Călin Cosmin Repciuc, Ioana A. Matei, Nicodim I. Fiț, Sanda Andrei, Raluca Marica, Bianca Nausica Petrescu, Bogdan Crișan, Ovidiu Aghiorghiesei, Ioana Codruța Mirică, Dragoș Apostu, Codruța Saroși, Florin Onișor, Evelyn Vanea, Simina Angela Lăcrimioara Iușan, Giorgiana Corina Mureșan, Ana-Maria Condor, Emilia Oprița, Luciana-Mădălina Gherman

**Affiliations:** 1 Department of Oral Health, Iuliu Hatieganu University of Medicine and Pharmacy, Str. Victor Babes 15, 400012 Cluj-Napoca, Romania; petrescu.bianca@umfcluj.ro (B.N.P.); aghiorghiesei.ovidiu@umfcluj.ro (O.A.); mirica.ioana@umfcluj.ro (I.C.M.); vanea.evelyn@elearn.umfcluj.ro (E.V.); iusan_simina_angela_lacrimioara@elearn.umfcluj.ro (S.A.L.I.); muresan_giorgiana_corina@elearn.umfcluj.ro (G.C.M.); ana.mari.condor@elearn.umfcluj.ro (A.-M.C.); constantin_ana_emilia@elearn.umfcluj.ro (E.O.); 2 Department of Surgery Anesthesiology and Intensive Care, Faculty of Veterinary Medicine, University of Agricultural Sciences and Veterinary Medicine, 400372 Cluj-Napoca, Romania; calin-cosmin.repciuc@usamvcluj.ro; 3 Department of Microbiology, Immunology and Epidemiology, University of Agricultural Sciences and Veterinary Medicine, 400372 Cluj-Napoca, Romania; ioana.matei@usamvcluj.ro (I.A.M.); nfit@usamvcluj.ro (N.I.F.); sandrei@usamvcluj.ro (S.A.); raluca.marica@usamvcluj.ro (R.M.); 4 Departament of Maxillofacial Surgery and Oral Implantology, Iuliu Hatieganu University of Medicine and Pharmacy, Cardinal Iuliu Hossu nr. 37, 400012 Cluj-Napoca, Romania; crisan.bogdan@umfcluj.ro (B.C.); florin.onisor@umfcluj.ro (F.O.); 5 Departament of Orthopaedics and Traumatology, Iuliu Hatieganu University of Medicine and Pharmacy, Traian Mosoiu nr. 47–49, 400012 Cluj-Napoca, Romania; apostu.dragos@umfcluj.ro; 6 Institute of Chemistry Raluca Ripan, Department of Polymer Composites, Babes-Bolyai University, 400294 Cluj-Napoca, Romania; liana.sarosi@ubbcluj.ro; 7 Experimental Centre of University of Medicine and Pharmacy Iuliu Hatieganu, 400349 Cluj-Napoca, Romania; luciana.gherman@umfcluj.ro

**Keywords:** periodontitis, membrane, gentamicin, nano-hydroxyapatite, drug delivery system

## Abstract

The aim of this research was to validate the use of a gentamicin (GEN) and nano-hydroxiapatite (nHAP)-loaded polycaprolactone nanostructured membrane (NM) as an innovative, highly efficient, low-cost treatment for periodontitis. We conducted an in vivo study on Wistar rats, in which we induced periodontitis by placing silk ligatures around the first right and left upper molars. The subjects were divided into three groups; the first group received no periodontal treatment, the second group received open flap debridement, and the third group received open flap debridement, together with the positioning of the GEN and nHAP-loaded nanostructured membrane as a treatment. The extent of periodontal regeneration was assessed by the periodontal pocket depth, bleeding on probing, tooth mobility, dental plaque, microbiological analysis, concentration of MMP-8 in saliva, plasma levels of CRP, and histological analysis. The results showed that using open flap debridement with the NM is more efficient, and it significantly reduces the probing depth, extent of bleeding on probing, dental mobility, bacterial plaque, and pathogenic flora. The concentrations of MMP-8 and CRP decrease. The histological analysis demonstrated that NM leads to bone regeneration. Our study indicates that gentamicin and nano-hydroxyapatite embedded in the fiber of the biodegradable membranes might be a promising therapeutic option for periodontitis treatment.

## 1. Introduction

Periodontitis (P-D) is a chronic immunoinflammatory disease of the periodontium with a high incidence, characterized by the progressive loss of periodontal ligament and gingival tissue, and also the loss of the adjacent supporting alveolar bone [1]. This disease not only has a significant impact on human health, but in the USA, the annual cost of periodontal therapy is estimated to be over USD 14 billion [2]. Furthermore, systemic diseases, such as rheumatoid arthritis [3], cardiovascular complication [4], and adverse pregnancy outcomes [5], have been associated with periodontitis.

Complex subgingival biofilms that contain several likely periodontal pathogens are responsible for initiating the chronic inflammation of the periodontium. Opportunistic pathogens of the oral cavity, such as *Porphyromonas gingivalis* (*P. gingivalis*), and a portion of the Gram-negative anaerobic commensal microbiota are generally a part of this biofilm [6]. Polymorphonuclear cells (PMNs) release destructive reactive oxygen species (ROS) like superoxides via respiratory bursts [7,8,9], proteinases, and other factors that can harm the host tissues [10,11,12] as a response to periodontal pathogens. Further, oxidative damage to the periodontal ligaments and gingival tissue is induced by these molecules, which also elicit bone resorption [10,13,14,15].

The levels of a broad variety of biomolecules and numerous proinflammatory cytokines that contribute to the disease, such as interleukin (IL-1β and IL-6) and tumor necrosis factor (TNFα), have been reported to be raised in patients with periodontitis [16,17,18]. Interleukins (IL-1, IL-6, and IL-8), tumor necrosis factor (TNFα), matrix metalloproteinases (MMP-8 and MMP-9), and tissue inhibitors of metalloproteinase are the most significant salivary inflammatory indicators associated with oral diseases [19].

Following periodontal therapy, the levels of these proinflammatory molecules are frequently reduced [20,21].

In light of the impact of this disease on oral and general health, a massive amount of research has been conducted in order to develop a new therapeutic approach. Root scaling and open flap debridement are widely used nowadays in clinical practice [22,23,24]. In any case, using these procedures, the new attachment achieved is often a result of the repair of the long junctional epithelium, with little or no new connective tissue attachment and cementum formation [25].

On the other hand, guided tissue regeneration (GTR), which uses a barrier membrane to prevent the epithelial cells and gingival tissue from reaching the exposed root surface, has been demonstrated to regenerate the tooth-supporting tissues, including the periodontal ligament, cementum, and new alveolar bone [26,27]. The ideal properties of the GTR membrane are that it eliminates the unwanted proliferation of epithelial cells into the defect, protects the underlying blood clot, and disintegrates in proper time in order to provide space for the cementoblasts, osteoblasts, and periodontal ligament cells to repopulate the root surface [28,29].

The traditional GTR membrane has been used primarily as a barrier to prevent epithelial cells from entering the defects prior to new bone formation. Recently, functional drug delivery membranes have been developed that control the release of drugs or growth factors to promote new bone regeneration [30,31,32]. The controlled and sustained delivery of antibiotics and/or growth factors has become a promising method to control the disease and improve periodontal remodelling.

Systemic drug administration has limited efficacy due to low local drug concentrations [33] and the development of antibiotic resistance [34] and is associated with systemic side effects, such as myelosuppression, drug-induced hepatitis, or nephrotoxicity [35]. Short delivery times, higher drug concentrations at the site of infection, and the elimination of systemic side effects make the local administration of antibiotics indicated [36]. Antibacterial biomaterials represent the broadest group of anti-infective biomaterials and are, therefore, of great interest in the fight against P-D.

A new generation of nanostructured barrier membranes (NMs) with improved properties is under development in order to improve cell proliferation, differentiation, migration, and adhesion, thereby promoting regenerative outcomes [37,38,39], and provide local antibiotic therapy. Combining a locally targeted antibiotic therapy with resorptive NMs using local regenerative strategies is an ideal therapy with predictable long-term outcomes.

The aim of this study was to explore a dual-release system of periodontal regeneration in an animal model using a new biodegradable NM made from poly(e-caprolactone) (PCL), with gentamicin (GEN) and nano-hydroxyapatite (nHAP) embedded in the fiber, for controlled release, obtained through an electrospinning process, as an innovative, highly efficient, low-cost treatment for P-D. This membrane was prepared and characterized by Mirica C. et al. [40].

## 2. Materials and Methods

### 2.1. The Method of Obtaining the NM

The obtaining of the ceramic/drug/polymer mixture started by dissolving GEN in a solvent solution of methanol/chloroform (3:1), which was stirred for 5 h with a magnetic bar, and sonicated for 30 min. The PCL was added, and the agitation process was repeated. All materials were acquired from Sigma-Aldrich, Darmstadt, Germany. After adding the nHAP nanoparticles, which were obtained in our lab [41], a sonication process took place using ELMA Elmasonic P70H (Elma Electronic Inc., Fremont, CA, USA) for 30 min, power 90%, frequency 80 KHz, at room temperature followed by another stirring for 14 h and sonication for 30 min. The electrospinnning process was completed with an experimental machine from Raluca Ripan, Institute of Research in Chemistry, Cluj-Napoca, Romania. The electrospinning parameters were voltage: 15–25 kv, flow rate: 2.5 mL/h, and distance between the tip of the needle and the collector: 31 cm. The obtained NMs (PCL-15%nHAP-2%GEN) were kept in a desiccator before testing for 48 h.

### 2.2. In Vitro Assays of the NM

The NM showed antibacterial activity in the disk diffusion assay against *S. mutans*, *S. aureus,* and *P. aeruginosa*, with inhibition zones of 0.78 cm, 1.36 cm, and 0.8 cm. The cytotoxicity analyzed through the MTT assay at 1 and 5 days showed a cell viability of 126.39% and 121.84%. The GEN release occurred in two phases: the burst release (first 6 h) of 424.8 ppm (53.63%) GEN, and a second phase (12 h) releasing 375.68 ppm (46,37%) GEN. The degradation rate was established by comparing the initial weight of the samples with the weight at 30 days and the weight loss reached 0.0004 g. Regarding the bioactivity, the hydroxyapatite formations were evidenced through SEM/EDX analyses after storing the samples in simulated body fluid for 21 days.

### 2.3. Rat Periodontal Defect Model Preparation

**Figure 1:**
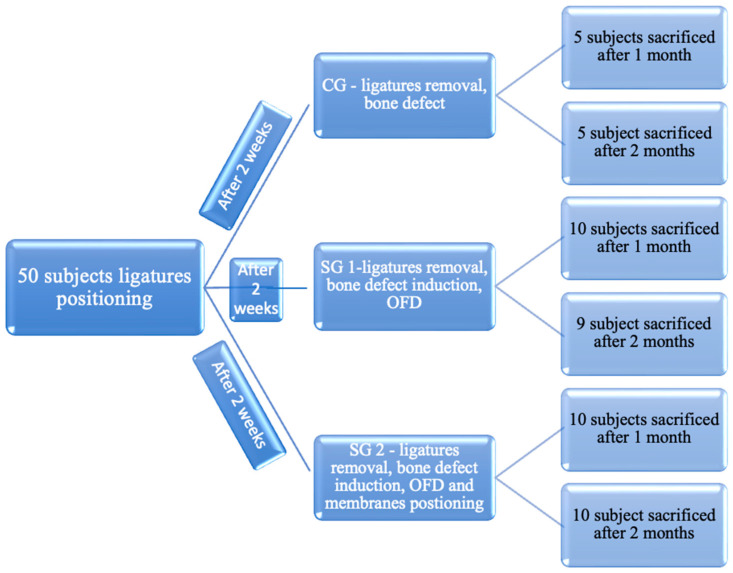
The division of the animal subjects in the study.

The study was authorized by the Medical Ethics Committee of The University of Medicine and Pharmacy “Iuliu Hatieganu”, Cluj-Napoca (Permit Number: 341 from 7 November 2022). A total number of 50 Wistar rats were enrolled in this study; one subject died. The rats were housed for acclimatization for about 1 week and then randomly assigned into the three groups. Subjects were divided into 3 batches: control group (Batch I) (10 subjects), study group 1 (Batch II) (19 subjects), and study group 2 (Batch III) (20 subjects) (Figure 1).

The experimental protocol was designed in three steps.

**Figure 2:**
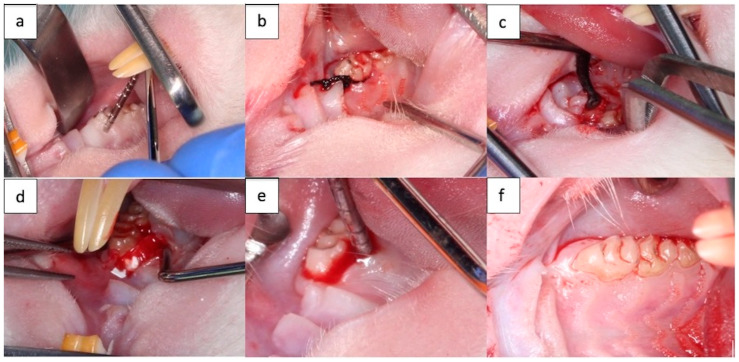
(**a**) Initial clinical evaluation; (**b**) application of the silk ligature; (**c**) open flap debridement; (**d**) membrane placement; (**e**) clinical assessment at 2 months of Batch I; (**f**) clinical assessment at 2 months of Batch III.

Step 1 consisted in the initial evaluation of specimens and application of the silk ligature (T0) (Figure 2a,b). In Step 2 (after 2 weeks), evaluation of the induced periodontal inflammation, ligature removal, and treatment application were conducted (Figure 2c,d). In Step 3 (at 1 month or 2 months after ligature removal), after clinical evaluation of therapeutic outcomes (Figure 2e,f), animals were euthanatized to collect tissue specimens for histological examination.

To create a clinically relevant periodontitis-associated bone defect model, the surgical periodontal defects were made by modifying the method reported by Nagata et al. [42] in molar teeth of rats with induced periodontitis.

Rats were anesthetized with an intraperitoneal injection of 0.1 mg/kg 10% Ketamine solution (Vetaketam, Przedsiębiorstwo Wielobranżowe VET-AGRO, Lublin, Poland) and 0.5 mg/kg of 2% Xylazine solution (Xylazin Bio, Bioveta, Ivanovice na Hane, Czech Republic). After that, they were placed on the operation table in horizontal supine position, which allowed access to place silk circumdental submerged ligatures at the level of the first upper right and left molars of all 49 subjects, obtaining 98 treatment sites. Ligatures were placed with the purpose of promoting plaque retention and subsequent local inflammation [43]. The application of the ligatures was performed under 2.5× magnification, using dedicated microsurgical instruments. Two weeks after, ligatures were removed, a palatal flap was elevated, and a bone defect (1 × 1 × 1 mm^3^ L W D) was prepared using a dental drill. The critical bone defect was located on the palatal bone plate of the first upper right and left molars. For subjects from the control group, after the bone defect induction flap was sutured, for subjects from study group 1, open flap debridement was performed (Figure 2c), and for subjects from study group 2, open flap debridement was associated with covering of the exposed palatal aspect with the new biodegradable NM from PCL, with GEN and nHAP embedded in the fiber membrane (Figure 2d).

All efforts were made to minimize the suffering of the animals. The animals were closely monitored for the entire length of the study, focusing on infection prevention and analgesic therapy.

One month after ligature removal was performed, half the number of subjects from each batch were euthanized (further named as Group 1 (Gr. 1)), and 2 months later, the other half (further named Group 2 (Gr. 2)).

### 2.4. Clinical Observation

The extent of wound healing was assessed visually by determining periodontal pocket depth, bleeding on probing, tooth mobility, and presence of dental plaque at the moment of ligature placement (T0), ligature removal (TI), and euthanasia (T2) (Figure 2e,f). Periodontal pocket depth was established using a periodontal probe, and probing was performed on the palatal aspect of the first right and left molar at the middle mesio-distal distance. Bleeding on probing was recorded as 0—absent, 1—bleeding on probing, 2—tendency to spontaneous bleeding. Mobility was scored as 0—absent, 1—low buccal lingual mobility, 2—moderate buccal–lingual and mesio-distal mobility, 3—severe axial mobility. Additionally, body weight was closely monitored.

### 2.5. Microbiological Analysis

The samples for the microbiological examination were collected from the crevicular sulcus at times T0, T1, and T2. Samples were collected from the level of the gingival sulcus using an endodontic absorbent paper cone. Each sample was added in 1 mL saline, being further diluted in 10-fold serial dilutions. Diluted samples were inoculated on 5% Columbia Blood Agar (Biomaxima, Lublin, Poland) and incubated up to 72h in anaerobic conditions (Anaerocult^®^ Jar, Anaerocult^®^ A, Millipore, Merck KGaA, Darmstadt, Germany). The total number of germs (NTG) was calculated based on the number of Unit Forming Colony (UFC) in a 1 mL diluted sample. For evaluation of the differences in NTG, three intervals were considered: <1000 UFC/mL (scored <1), between 1000 and 3000 UFC/mL (scored 1–3), and >3000 UFC/mL (scored >3). For the species identification, a sub-culture of each colony type (pure culture) was obtained in the same conditions as described above. Bacteria isolates were identified using the Vitek^®^ 2 compact 15 system (bioMérieux, Marcy l’Etoile, France).

### 2.6. Blood and Saliva Samples

Stimulated saliva was collected at T0, T1, and T2 for salivary MMP-8. In order to analyze the concentration of MMP-8 in saliva, an in vitro enzyme linked immunosorbent assay was used (kit RayBio, Norcross, GA, USA). Analysis involved the use of antibodies specific for MMP-8. Seven standard solutions in total were made in order to obtain the standard curve: 40, 13.3, 4.44, 1.48, 0.49, 0.16, and 0.05 ng/mL. The equation of the standard curve was then applied to determine the MMP-8 levels for each saliva sample.

Blood samples from the retro-orbital artery were collected at T0, T1, and T2 so that Plasma Levels of CRP could be determined. The samples were analyzed using ELISA Kit (ABclonal, Woburn, MA, USA).

The C-reactive protein in rats (CRP) is 100 times higher in concentration than in humans, under basal conditions, i.e., 300–500 mg/L. Also, it is an inflammation marker [44].

### 2.7. Histological Analysis

Rats were euthanized at 1 and 2 months post-surgery, and maxillaries with defects were collected and fixed in 10% formalin solution. All samples were decalcified with 10% hydrochloric acid decalcifier TBD-1 (Epredia^TM^, Brussels, Belgium) for 1 week and routinely dehydrated, embedded, and cut into 4 μm sections in the vestibulo-lingual direction. Hematoxylin-eosin (H&E) staining was conducted.

### 2.8. Statistical Analysis

In order to perform the statistical analysis, we used Microsoft Excel Spreadsheet (Office 2021) for the descriptive statistics and the Shapiro–Wilk normality test to determine the type of data distribution [45]. The analysis of the normal data from a group was carried out with a *t*-test for 2 dependent means and the analysis between groups and batches was carried out with a *t*-test for 2 independent means. For data with non-normal distribution, the Mann–Whitney U test was used along with Chi-square statistics for categorical data, with significance level *p* = 0.05 [46].

## 3. Results

### 3.1. Clinical Assessment

#### 3.1.1. The Effects of Ligature Placement (Procedure 1)

The placement of the ligatures leads to modification in weight (g), probing depth (mm), bleeding on probing, dental mobility, and bacterial plaque. For bleeding, dental mobility, and bacterial plaque, both the frequency of occurrence and the abundance/severity of the condition (*—low, **—medium, ***—high) were considered (Table 1).

The placement of the ligatures leads to a decrease in the mass and an increase in the probing depths. It also led to bleeding during probing, dental mobility, and bacterial plaque, which were initially absent. The decrease in mass was statistically significant (*p* < 0.0001) and was equal to about 5.2% on average. The probing depth increase of 5.26 times was also statistically significant (*p* < 0.0001)

Implicitly, bleeding on probing (91.83%), microbial plaque (75.51%), and tooth mobility (73.46%) were observed. From the point of view of gravity, the effect of the ligature is greater on dental mobility and bacterial plaque; thus, 56.12% of the rat subjects had dental mobility and 59.18% had bacterial plaque present at T1. Also, 17.34% of rats had severe forms of mobility and 16.32% had bacterial plaque.

#### 3.1.2. The Effects of the Intermediate Procedure Applied after Removing the Ligature

##### Changes to the Weight of the Subjects

Between the moment of removing the ligature and the application of the next procedure until the moment of euthanasia, the weight increased. This increase was influenced by both the type of intermediate procedure and the duration between it and the euthanasia. In Batch II, 78% of the rats, and in Batch I, 60% of the rats suffered various pathological complications, compared to Batch III, where only 20% had complications. The complications were right/left abscess, fistula, halen, exposed furcation, trismus, pus, and necrosis. These complications are reflected in the evolution of the rats’ weight and weight correlation, especially in short-term evolution (Table 2).

In both groups, Batch III developed better than Batch II and Batch I. The increase in weight in Batch III Gr. 2 is statistically significantly higher than the increases in Group 2 in Batch II and Batch I. There are no statistical correlations between Batch II and Batch I. In each Batch, the weight at 2 months (Gr. 2) was statistically significantly higher than at 1 month (Gr. 1).

##### Probing Depths (PD)

**Figure 3:**
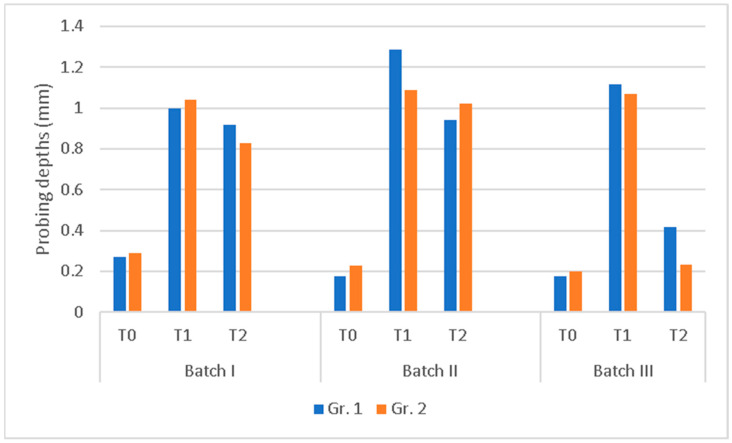
Probing depth variation during the 3 procedures.

Probing depths increased after placing the ligature. The subsequent treatment procedure led to a decrease in values, and the decrease was influenced by the type of procedure and the duration of the action (Figure 3, Table 3).

Batch III had lower probing depths at 1 month and 2 months than Batch I and II. In Gr. 1, the final probing depths represented 37.5% of the values before process 2 and were 2.3 times higher than the initial ones. In Gr. 2, they represented 22.4% of the intermediate ones and were 1.2 times higher than the initial ones. Based on these results, we can consider that a period of approximately 2 months is enough for a significant reduction in the probing depths.

In Batch III, the probing depths decreased significantly, and the healing was better, so the membrane was already effective after 1 month. This was also true in Batch II Gr. 1, where the probing depths decreased as a result of the curettage, an effect that then disappeared at 2 months.

Batch III had the best healing compared to other batches for both subjects sacrificed at 1 month and at 2 months, the differences being statistically significant.

##### Bleeding on Probing, Dental Mobility, Bacterial Plaque

**Figure 4:**
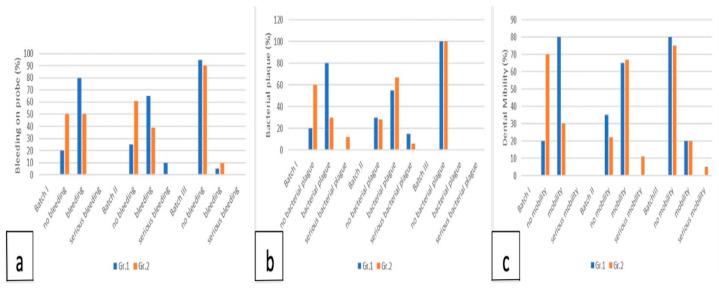
(**a**) Bleeding on probing at T2; (**b**) bacterial plaque at T2; (**c**) dental mobility at T2.

These parameters decreased from T1 (the removal of the ligature and the application of procedure 2) until T2 (the time of sacrifice), both in terms of the number of affected subjects and the severity of the condition (Figure 4a–c).

In Batch III, the absence of bleeding was at 90–95% in both groups, the mobility at 1 month was absent in 80% of the subjects, and at 2 months, it decreased to 75%. The bacterial plaque completely disappeared already at 1 month.

Batch I, to which no procedure was applied after removing the ligature, had a slow evolution, reaching the absence of bleeding and mobility after 2 months in only 40% of cases, and the absence of plaque in 60% of cases.

Batch II reached at 1 month the absence of bleeding in 40% of the cases, mobility in 35% of them, and bacterial plaque in 30%. After 2 months, the absence of bleeding increased to 55% of cases, mobility instead decreased to 20% of cases, and bacterial plaque reached 27%.

The comparison of Batch III with Batch I and Batch II showed a considerable improvement in both groups in Batch III, the differences being statistically significant (Gr. 1 χ^2^ = 24.75, *p* < 0.0001, Gr. 2 χ^2^ = 6.48, *p* = 0.03915).

Regarding dental mobility, it had a behavior similar to bleeding on probing, meaning that there was a statistical correlation between Batch III and Batch II for both groups, respectively, Batch III and Batch I for Gr. 1, with the same observation, the lowest dental mobility being registered in Batch III and being statistically significant (Gr. 1 χ^2^ = 12.6, *p* = 0.0018, Gr. 2 χ^2^ = 18.683, *p* = 0.00088).

As for the bacterial plaque, at both 1 month and 2 months, there was a significant difference between Batch II and III. More exactly, in Batch III, the plaque was resolved in a single month, while in Batch II, there were only about 27–30% of cases without bacterial plaque. On the other hand, in Batch I, the absence of bacterial plaque increased from 20% at 1 month to 60% at 2 months, without any correlations with other batches.

### 3.2. Microbiological Analysis

#### 3.2.1. The Effects of Ligature Placement on NTG and Microbiological Flora

**Figure 5:**
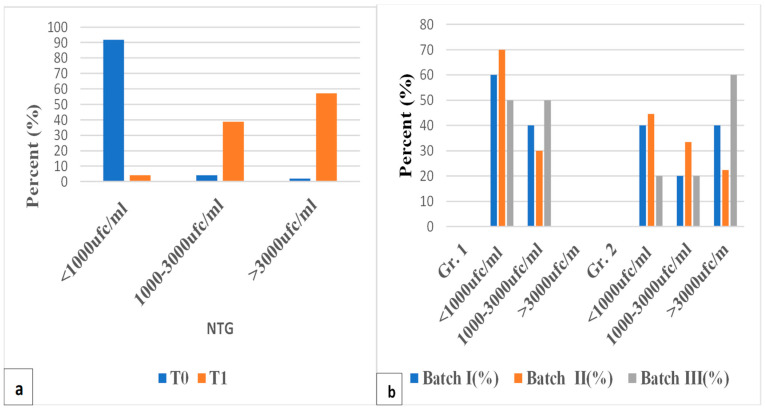
(**a**) The effect of the ligatures on NTG; (**b**) the effect of the second procedure at T2 for all three batches.

NTG values increased significantly (>3000 UFC/mL) at T1. The total flora had an increase of 1.56 times at time T1, compared to T0, and the total pathogenic flora was 1.895 times. Also, the abundance of each pathogenic organism experienced major changes according to Figure 5a and Table 4.

At T1, the frequency of occurrence of *Staphylococcus aureus* increased (χ^2^ = 10.227, *p* = 0.001384) and was statistically significant, while *Actinomyces wessie* (χ^2^ = 24.81, *p* < 0.0001) decreased significantly. According to the abundance, *Streptococcus mutans* had the highest abundance, about 25% (high abundance ***) (χ^2^ = 44.219, *p* < 0.0001).

#### 3.2.2. The Effects of the Intermediate Procedure Applied after Removing the Ligature on NTG and Pathogenic Flora

##### NTG

In all batches, NTG values >3000 UFC/mL were no longer present in Gr. 1. At T2, all intermediate procedures led to a decrease in NTG values.

It is worth noticing that the values <1000 UFC/mL are higher in Batch II than Batch III, and the intermediate values are lower in Batch II than the other batches; thus, simple curettage seems more useful at 1 month. In Gr. 2, a smaller decrease in NTG is observed in Batch III compared to Batch II, the values being slightly reduced compared to T1 (membrane maintained NTG) (Figure 5b).

##### Pathogenic Flora

**Figure 6:**
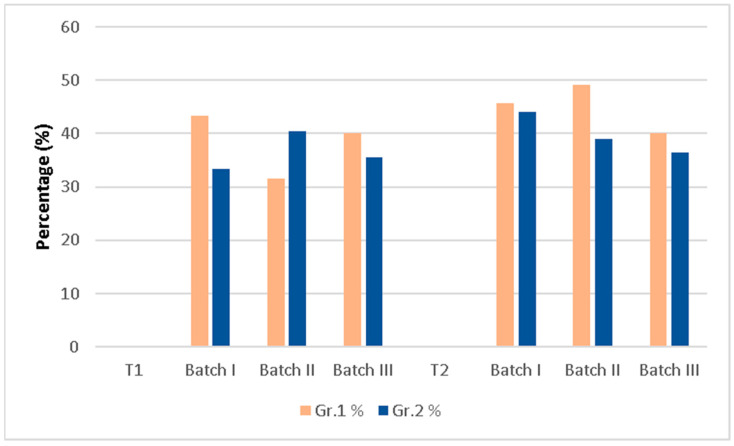
Pathogenic flora relative to the total number of germs.

The group of pathogenic germs compared to all measured flora is presented in Figure 6.

In Gr. 1 of Batch II, the number of pathogenic germs compared to the total number of germs was statistically significantly different at T2 (49.2%), being higher than at T1 (31.6%), χ^2^ = 3.9122, *p* = 0.04793, while in Batch III, there are no statistically significant differences, the pathogenic germs being 40% at both T1 and T2, so the membrane with gentamicin seems to be more effective in the short term.

In Gr. 2, there were no statistical differences in any batch and there were no statistical differences between the batches at the final moment.

In Gr. 1, Streptococcus mutans had a low frequency in Batch III compared to Batch II (χ^2^ = 8.1027, *p* = 0.00442), showing that the gentamicin incorporated into the membrane was effective at 1 month on Streptococcus mutans.

In Gr. 2, Streptococcus mutans was less common in Batch III than in Batch II (*p* = 0.032509).

The Streptococcus mutans frequency remained approximately the same in both groups of Batch III at the final moment T2; on the other hand, in Batch II, the frequency in Gr. 1 was higher compared to Gr. 2.

### 3.3. Blood and Saliva Sample Results

The experimental data obtained for the concentrations of MMP-8 and CRP are included in a wide range of values.

#### 3.3.1. The Effect of the Ligature on MMP-8 and CRP

At ligature removal (T1), the concentrations of MMP-8 and CRP were higher than those at the beginning of the experiment, all increases being statistically significant (Table 5).

#### 3.3.2. The Effect of the Intermediate Procedure on MMP-8 and CRP

The MMP-8 concentrations between the second and third procedure are presented in Table 6.

##### Correlation for MMP-8

After procedure 2, the concentrations of MMP-8 decreased in each batch. The highest decrease occured after one month, respectively, in group 1. In group 1, Batch III, the concentration decreased 5.22 times, the decrease being statistically significant (*p* = 0.00047); compared with Batch II, it was 3.16 times (*p* = 0.02414), and compared with Batch I, it was 1.61 times. In group 2, the changes were not statistically significant.

The CRP average concentration values decreased at T2 compared to T1 in all groups and batches (Table 7).

##### Correlation for CRP

The concentration was in each batch lower at T2 than T1, and the highest decrease was in group 1, Batch III. A statistically significant correlation was obtained for the concentration differences between group 1 of Batch II and Batch III, the decrease being 2.21 times greater in the case of Batch III compared to Batch II (*p* = 0.02275).

### 3.4. Histological Analysis

#### 3.4.1. Batch I

##### Gr. 1

**Figure 7:**
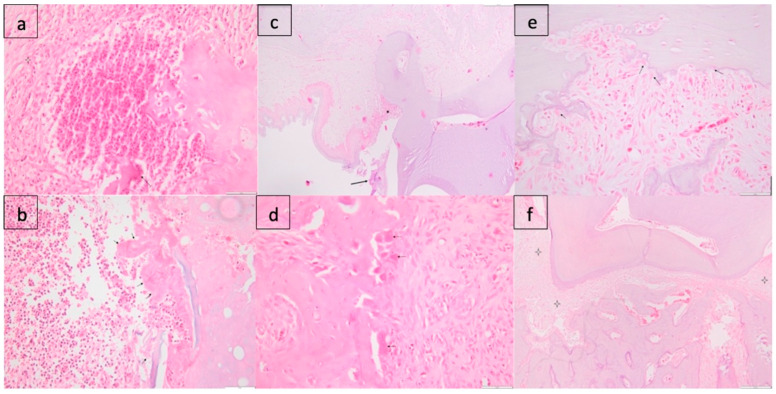
(**a**) Batch I, Gr. 1: bone lysis (arrow), abscess, granulation tissue (star); (**b**) Batch I, Gr. 2: bone fragment, with severe osteolysis (arrows), marked inflammatory infiltrate and hemorrhage; (**c**) Batch II, Gr. 1: bacterial plaque (arrow), periodontal gingival inflammation (star); (**d**) Batch II, Gr. 2: foci of bone remodelling, incipient—the presence of osteoclasts (arrows) on the surface of bone lamellae, accompanied by granulation tissue; (**e**) Batch III, Gr. 1: numerous osteoblasts (arrows); (**f**) Batch III, Gr. 2: periodontal fibrosis (stars).

The subepithelial conjunctive tissue of the gingiva is diffusely infiltrated by a moderate to severe inflammatory infiltrate, composed predominantly of polymorphonuclear cells. There is congestion and a moderate interstitial edema. Focally, the overlying epithelium (in two individuals) is lost and replaced by fibrin, hemorrhage, and nuclear debris of inflammatory cells. Alveolar bone presents osteolysis, and there are a high number of active osteoclasts at the surface of bone lamellae. Multifocally, there are many bone fragments detached, surrounded by inflammatory cells (PMN) and showing necrosis (Figure 7a).

##### Gr. 2

There is moderate to severe multifocal infiltration with mononuclear cells (macrophages, lymphocytes) in the gingival tissues, accompanied by granulation tissue. The overlying epithelium is moderately hyperplastic, showing hyperkeratosis.

Alveolar bone is severely affected, and shows osteolysis, and a severe diffuse inflammatory reaction with mononuclear and polymorphonuclear cells (Figure 7b).

#### 3.4.2. Batch II

##### Gr. 1

The pathological changes are similar to those observed in group I individuals but decreased in severity. There is a subepithelial inflammatory infiltrate, predominated by PMN, accompanied by moderate congestion, edema, and a loose granulation tissue, with numerous small-caliber blood vessels and fibroblasts. In some individuals, we can observe multiple bacterial colonies between the tooth and the junctional epithelium. Regarding the alveolar bone, the presence of periosseous granulation tissue and also early bone remodelling phenomena can be observed through the osteoblastic reaction on the surface of the bone lamellae (Figure 7c).

##### Gr. 2

There is mild to moderate hyperplasia of the surface epithelium, with slight hyperkeratosis. A moderate inflammatory infiltrate with mononuclear cells is noted in the alveolar bone. The osteoblastic and osteoclastic activity is increased,

Also, there is diffuse, extensive fibrosis adjacent to the alveolar bone, diffusely infiltrated by a mild mononuclear inflammatory infiltrate (Figure 7d).

#### 3.4.3. Batch III

##### Gr. 1

In Batch III, inflammatory phenomena reduced in intensity, and proliferation of fibrous tissue and bone regeneration are observed. Regarding the gingival lesions, a minimal inflammatory infiltrate is noted, dominated by mononuclear cells, subepithelial fibrosis, and gingival hyperplasia. Bone regeneration is observed, as well as numerous osteoblasts on the surface of the bone tissue. A focus of pyogranulomatous osteomyelitis was identified in one individual, with an inflammatory infiltrate predominated by polymorphonuclear cells and macrophages, with bone lysis (Figure 7e).

##### Gr. 2

There is moderate peri-alveolar fibrosis. Intense bone remodelling is demonstrated by the increased number of activated osteoclasts, along with numerous osteoblasts. No bone fragments or bacterial dental plaque were observed. There is minimal inflammatory reaction in the sub-epithelial connective tissue of the gingival, composed of mononuclear cells, accompanied by fibrosis (Figure 7f).

## 4. Discussion

The aim of our study was to validate a new drug delivery system for periodontal regeneration. The periodontal disease is a chronic disease of the periodontium that is characterized by the progressive loss of the gingival tissue, periodontal ligament, and the supporting alveolar bone. This disease has an impact on the general and oral health; thus, a lot of research has been carried out in order to develop new therapeutic approaches.

We conducted an in vivo study where periodontal disease was induced in rats by placing silk ligatures around the first upper molars and by inducing a surgical bone defect.

The placement of the ligature led to a statistically significant decrease in the weight, to a statistically significant increase in the probing depths, to an increase in NTG (statistically significant), and to the development of pathogenic flora (statistically significant), with a special emphasis on *Streptococcus mutans* and *Staphylococcus aureus* of the entire batch of 50 rats measured 2 weeks after the placement of the ligature. The ligature led to the appearance of bleeding on probing, tooth mobility, and bacterial plaque, these being initially absent. All this demonstrated the efficiency of the protocol used to induce periodontitis.

The curettage with the application of the membrane loaded with GEN and nHAP (Batch III) was the most effective in recovering the rats’ weight, this being already recovered in the case of Batch III after one month, compared to Batch II where the weight was recovered only after 2 months, while in Batch I, the subjects did not fully recover their initial weight.

The removed ligature combined with curettage and membrane placement was more efficient, reducing the probing depths significantly, while only the simple curettage did not considerably improve the final periodontal status, the control Batch I being the least improved.

Similarly, the healing was the most pronounced in Batch III in all the parameters: bleeding on probing, dental mobility, and bacterial plaque, with values that differ statistically significantly from those of Batch II and Batch I. In Batch III the bacterial plaque was absent already after 1 month after procedure 2, mobility was reduced to 75–80%, and bleeding disappeared in 90–95% of the subjects. Thus, the procedure implied that ligature removal, curettage, and membrane application (Batch III) accelerated the healing of bleeding, dental mobility, and bacterial plaque.

According to NTG, the simple curettage is more effective, and the more significant effect was observed after 1 month. On the other hand, the ratio between pathogenic flora and normal flora was more favorable in Batch III, at 1 month, being approximately constant, while in Batch II, a significant increase in the pathogenic flora was observed. The action of gentamicin on *Streptococcus mutans* in Batch III is noteworthy, its frequency being lower in both situations (Gr. 1 and Gr. 2).

MMP-8 and MMP-9 have been linked to periodontal disease in humans, according to a number of studies. In response to inflammatory conditions, neutrophils, endothelium and smooth muscle cells, and macrophages release neutrophil collagenase/MMP-8. Periodontitis-related gradual loss of attachment is correlated with salivary MMP-8 levels. As a result, MMP-8 has been suggested as a biomarker for early periodontitis diagnosis [18].

Based on the results of the present study, it can be concluded that after the removal of the ligature, the concentration of MMP-8 decreases, being significant at 1 month, both for the simple curettage (2.93 times) and curettage plus membrane (3.99 times). On the other hand, at 2 months, the values for Batch III were lower at T2 than T1, this meaning that in the longer term, the curettage plus membrane is more effective in terms of MMP-8 concentration.

Each intermediate procedure led to a decrease in CRP concentrations, the decrease being more significant in Batch III after 1 month, showing that curettage with membrane application is more effective. It should be noted that the average values of concentrations at 2 months are within the limit of normal values in both Gr. 1 and Gr. 2.

The histological analysis proves that simple curettage only produces healing by gingival hyperplasia, at the same time maintaining a moderate inflammatory infiltrate, while the gentamicin-loaded membrane used after curettage leads to bone regeneration and gingival hyperplasia with minimal inflammatory infiltrate.

The use of membranes for the regeneration of periodontal structures is a current topic of interest, with many researchers trying to propose membranes with different components and active substances.

Similar to the present study, Ning Li et al. (2019) developed a chitosan membrane containing polyphosphoester and minocycline hydrochloride that was used in an in vivo study in a periodontal defect model in rats (12 rats) and proved that the membrane was effective in alleviating ligature-induced periodontitis bone loss and partly restored the bone mass in periodontitis [47].

In another study, Amir Nasajpour et al. (2018) evaluated a polycaprolactone composite membrane containing zinc oxide nanoparticles with a rat periodontal defect model (10 rats) and confirmed that the membrane exerts both antibacterial and osteoconductive properties, having a great potential for tissue engineering [48].

Xuezhe et al. (2020) developed a multifunctional periodontal membrane prepared by electrospinning biodegradable polymers with magnesium oxide nanoparticles and assessed its effects in vivo on a rat periodontal defect model (24 rats) and showed that the membrane effectively guided periodontal tissue regeneration [49].

These three studies managed to prove the effectiveness of their membrane but were carried out on a small number on subjects. In addition, the evaluation methods were different and less complex compared to the present study. Ning Li et al. (2019) evaluated the membrane in vivo by measuring ABC-CEJ distances at six anatomical sites in two maxillary molars, including three points at the palatal and buccal sides of the rats [47]. Amir Nasajpour et al. (2018) evaluated the effect of the membrane by using micro-CT analysis to examine the amount of bone regeneration, while Xuezhe use microcomputed tomography to scan harvested samples and measured the distance between the cementoenamel junction and alveolar bone crest in order to assess the vertical bone loss [48].

More recently, Shuangshuang Ren et al. (2022) proposed a fibrous membrane containing cerium oxide nanoparticles for periodontal tissue engineering and conducted an in vivo study in rat cranial defect [50]. Even if they have demonstrated the effectiveness of the membrane for bone regeneration, by using it at the level of cranial bone, they did not consider or evaluate the pathogenic flora present in periodontitis, which might have an impact on the effect of the membrane.

Similarly, Lingling Shang et al. (2021) used a dimethyloxalylglycine and nanosilicate polylactic-co-glycolic acid fibrous membrane in a rat periodontal defect model [51]. The study showed that the membrane induced osteogenesis and angiogenesis and regenerated the cementum–ligament–bone complex, but the immunomodulation and anti-infection effects of the fibrous membranes were not evaluated. While these studies show the effectiveness of their tested membranes in periodontal regeneration, they do not evaluate the effect of the membranes on the pathogenic flora involved in the periodontal disease. Since periodontitis is an infectious disease, this could be considered an important limitation of the above-mentioned studies. In the present study, the nHAP membrane and GEN was tested in induced periodontitis in rats and proven to be effective in periodontal regeneration and reduction in pathogenic flora.

Our results are in line with those obtained by the previously mentioned authors. All described membranes prevented cell and tissue penetration, providing physical barrier function and hindering fibroblast penetration to periodontal defects. They proved that mechanical properties withstand the forces exerted by the covering flaps. The degradation rate of polymeric membranes is around 6 weeks, as needed for periodontal regeneration, impeding epithelial invagination and offering space for periodontal regeneration. Incorporating polymeric membranes of magnesium, nanosilica, or nHAP, we can stimulate bone regeneration through cell proliferation, and through the incorporation of magnesium oxide nanoparticles, zinc oxide nanoparticles, minocycline hydrochloride, or GEN and we can lower bacteria adhesion. All the above presented membranes proved to be a promising local delivery strategy of antibiotics and bone inducers.

Taking into account all previously mentioned aspects about these studies, we can state that our study is a complex in vivo study regarding the use of membranes in periodontal regeneration, by using 49 rat subjects and evaluating the effectiveness of the membrane by a large number of parameters, such as subject weight, probing depth, bleeding on probing, tooth mobility, bacterial plaque, histological analysis, blood samples (CRP), saliva samples (MMP-8), and bacterial flora. To our knowledge, this is the first membrane loaded with nHAP and GEN obtained through electrospinning tested in vivo in an animal model.

## 5. Conclusions

Combining all the results, the present in vivo study indicates that GEN and nHAP incorporated in biodegradable membranes might be a promising therapeutic option for periodontal disease treatment.

## Figures and Tables

**Table 1:** The effects of the ligatures on the rat subjects.

	T0	T1		*p*-Value
Weight (g) ± SE	307.55 ± 3.34	291.87 ± 4.42		*p* < 0.0001
Probing Depths (mm) ± SE	0.212 ± 0.01	1.1153 ± 0.02		*p* < 0.0001
Bleeding on Probing (No)	0	90 [89 *, 1 **]	91.83%	
Dental Mobility (No)	0	72 [55 *, 13 **, 4 ***]	73.46%	
Bacterial plaque (No)	0	74 [58 *, 14 **,2 ***]	75.51%	

*—low, **—medium, ***—high.

**Table 2:** Variations in the weight of the subjects (mass distributions are normal).

Average Values (g) ± SE	m1 (g) at T1	m2 (g) at T2	Correlation between Groups Correlation between Batches
Batch 1			BIII vs. BI Gr. 2 *p* = 0.044045
Gr. 1	299.6 ± 13.17	300.8 ± 10.16	*p* = 0.00843
Gr. 2	298.6 ± 12.44	343.2 ± 13.32	
Batch II			BIII vs. BII Gr. 1 *p* = 0.03689
Gr. 1	263.7 ± 11.16	286.1 ± 13.49	*p* = 0.002409
Gr. 2	301.4 ± 10.41	339.9 ± 8.85	
Batch III			B III vs. BII Gr. 2 *p* = 0.012545
Gr. 1	286.1 ± 7.71	317.1 ± 9.01	*p* = 0.000288
Gr. 2	310.0 ± 4.57	370.1 ± 8.52	

**Table 3:** Probing depths (PD) and correlations between batches.

Average Values (mm) ± SE	PD (mm) at T1	PD (mm) at T2	Correlation between Batches
Batch 1			B II vs. B I Gr. 2 *p* = 0.0294
Gr. 1	1.00 ± 0.01	0.92 ± 0.04	
Gr. 2	1.03 ± 0.02	0.83 ± 0.15	B III vs. B I Gr. 2 *p* = 0.0006
Batch II			B III vs. B I Gr. 1 *p* = 0.0004
Gr. 1	1.29 ± 0.08	0.94 ± 0.08	
Gr. 2	1.09 ± 0.03	1.02 ± 0.08	B III vs. B II Gr. 2 *p* < 0.0001
Batch III			B III vs. B II Gr. 1 *p* < 0.0001
Gr. 1	1.12 ± 0.04	0.42 ± 0.05	
Gr. 2	1.07 ± 0.047	0.24 ± 0.047	

**Table 4:** Effect of ligature on NTG and pathogenic flora.

NTG (×1000 UFC/mL) (No)	No at T0	No at T1	% at T0/T1	*p*
<1	46	2	91.84%/4.08%	*p* < 0.00001
1–3	2	19	4.08%/38.77%	χ^2^ = 73.2383
>3	1	28	2.04%/57.14%	
Pathogenic Flora (No)			Abundance (%)	
*Streptococcus mutans*	12 (total) 12 *	31 (total), 27 ***	28.7 25	χ^2^ = 44.219, *p* < 0.0001
*Staphylococcus aureus*	6 (total), 5 *	36 (total), 8 ***	33.34 7.41	χ^2^ = 24.81, *p* < 0.0001
*Actinomyces weissii*	25 (total) 24 *	11 (total) 8 ***	10.19 7.41	χ^2^ = 24.81, *p* < 0.0001

*—low, ***—high.

**Table 5:** Effect of ligature on MMP-8 and CRP concentration.

	Average Concentration at T0	Average Concentration at T1	*p*
MMP-8 ng/mL saliva	99.88 ± 6.93	293.95 ± 28.10	*p* < 0.0001
CRP μg/mL blood	271.86 ± 29.07	568.33 ± 65.91	*p* = 0.0027

**Table 6:** MMP-8 concentration at T1 and T2.

	Average Concentration at T1	Average Concentration at T2
Batch I		
Gr. 1	191.50 ± 88.50	118.65 ± 11.27
Gr. 2	193.28 ± 148.37	163.83 ± 11.27
Bath II		
Gr. 1	377.25 ± 274.54	119.12 ± 55.56
Gr. 2	332.32 ± 255.68	207.23 ± 41.21
Batch III		
Gr. 1	584.49 ± 264.39	112.41 ± 26.49
Gr. 2	325.38 ± 173.83	172.23 ± 141.51

**Table 7:** CRP concentration at T1 and T2.

		Average Concentration at T1 (μg/mL)	Average Concentration at T2 (μg/mL)
Batch I	Gr. 1	304.44 ± 63.03	295.55 ± 38.07
	Gr. 2	656.00 ± 200.7	572.00 ± 132.5
Batch II	Gr. 1	454.00 ± 305.42	370.55 ± 258.87
	Gr. 2	551.66 ± 118.98	515.55 ± 99.36
Batch III	Gr. 1	744.00 ± 17.28	558.80 ± 141.69
	Gr.2	559.00 ± 131.60	465.50 ± 87.65

## Data Availability

The original contributions presented in the study are included in the article, further inquiries can be directed to the corresponding author.
